# Adipokines and Cysteinyl Leukotrienes in the Pathogenesis of Asthma

**DOI:** 10.1155/2015/157919

**Published:** 2015-12-06

**Authors:** Michael J. Coffey, Barbara Torretti, Peter Mancuso

**Affiliations:** Division of Pulmonary and Critical Care Medicine, University of Michigan Medical Center, Ann Arbor, MI 48109, USA

## Abstract

*Background*. The prevalence of obesity has increased dramatically over the last decades, and its association with asthma is being increasingly recognized.* Aims*. Our hypothesis is that increased leptin and decreased adiponectin levels in obese subjects play a direct role in regulating inflammation in asthmatics. We wanted to examine the hypothesis that cysteinyl leukotrienes (cys-LT), inflammatory mediators that are regulated by adipokines, are involved in the pathogenesis of asthma.* Methods*. We studied a population of asthmatics and nonasthmatics, who in turn were divided into obese and nonobese categories. We examined leptin and its ratio to adiponectin, in asthmatics and nonasthmatics, with and without obesity. In addition, we measured cys-LT levels in exhaled breath condensate (EBC) and in peripheral blood monocytes (PBM) in these groups.* Results*. Leptin levels were increased in obese asthmatics compared to obese nonasthmatics. The leptin/adiponectin (L/A) ratio was higher in obese asthmatics compared to obese nonasthmatics. EBC cys-LT levels were elevated in asthmatics compared to nonasthmatics.* Discussion*. Proinflammatory adipokines, released from adipose tissue, may promote an asthma phenotype through enhanced cys-LT production that may result in more prevalent and difficult to control airway disease.

## 1. Introduction

Over the past 30 years there has been a dramatic increase in the prevalence in obesity in the US, from 18% in the 80s to approximately 35% in 2010 [1, 2]. In parallel, there has been an increase in the prevalence of asthma from 30.7 to 53.8 per thousand population between 1980 and 1994 [3]. A prospective epidemiologic study demonstrated a positive independent association between obesity and the incidence of adult onset asthma [4]. Likewise, using National Health and Nutrition Examination Survey data, Ma et al. have reported an independent association between obesity and atopic and nonatopic asthma [5]. Furthermore, obesity is independently associated with increased bronchial hyperresponsiveness [6]. Intervention with weight reduction improves airway narrowing and bronchial hyperresponsiveness [7]. Obesity is associated with a dose-dependent increase in the odds of incident asthma [8, 9].

Leukotrienes (LT) are potent lipid mediators of inflammation derived from arachidonic acid known to play a critical role in the pathogenesis of asthma. Arachidonic acid is acted upon by the enzyme 5-lipoxygenase to synthesize LTB_4_ and cysteinyl leukotrienes (cys-LT C_4_, D_4_, and E_4_). Both cys-LT and LTB_4_ are released by neutrophils, eosinophils, mast cells, peripheral blood monocytes, and macrophages and are involved in the pathogenesis of obstructive lung disease [10]. LT synthesis has been shown to be increased in peripheral blood leukocytes [11, 12], airway macrophages, and eosinophils [13] from asthmatics compared to cells from healthy controls. Asthma with obesity is often difficult to control and manifests steroid resistance [14]. Interestingly, obese subjects that had reduced responsiveness to inhaled corticosteroids with increasing body mass index (BMI) demonstrated a stable response to treatment with the LT modifier, montelukast [15]. Therefore, LTs may play a greater role in the pathogenesis of asthma in obese subjects compared to nonobese patients.

Obesity is a proinflammatory state associated with increased systemic cytokine levels such as tumor necrosis factor *α*, interleukin-1*β*, and interleukin-6 and other classical mediators produced by adipocytes termed adipokines [16]. Adipokines are hormones that display both pro- and anti-inflammatory properties. One such adipokine is leptin, a 16 kD protein synthesized by adipocytes and involved in the regulation of food intake and energy balance [17]. Levels of plasma leptin correlate with total body fat stores, being elevated in obesity and reduced in weight loss. Leptin has been shown to enhance proinflammatory cytokine expression, specifically to upregulate Th1 cytokines [18] and the eicosanoid, LT [19, 20]. In contrast to the proinflammatory effects of leptin, adiponectin is predominantly an anti-inflammatory mediator, mainly involved in glucose control and fatty acid metabolism, that is synthesized in adipose tissue and plasma levels of adiponectin are decreased in obesity.

A role for leptin in promoting pulmonary inflammation and bronchoconstriction has been demonstrated in murine models of obesity and asthma [21, 22]. However, data supporting a role for leptin in the prevalence or severity of asthma in adult humans, independent of BMI, are inconsistent [23]. Adiponectin has also been shown to play a role in animal models of asthma but, in contrast to leptin, this adipokine attenuates pulmonary inflammation [24, 25]. The impact of reduced serum adiponectin levels in humans with asthma is, like leptin, not clearly defined [23]. Preliminary data from our laboratory demonstrate increased plasma leptin levels in obese asthmatics compared to obese nonasthmatics. Leptin levels also tended to increase in asthmatics compared to nonasthmatics whether they were obese or nonobese. In view of the positive association between leptin and LT level, we examined cys-LT levels in the exhaled breath condensate (EBC) and peripheral blood leukocytes of asthmatics compared to nonasthmatics and obese compared to nonobese subjects.

## 2. Methods

### 2.1. Recruitment

All subjects were 18–65 years old with or without asthma, male and female, with ethnicity and race distribution of the community's population seen at the University of Michigan Health Centers. The nonasthmatic subjects were healthy volunteers without a history of lung disease. Fully informed consent was obtained from each subject before entry into the study. All procedures were approved by the Institutional Review Board. BMI calculations were determined by dividing the weight by the square of the height (kg/m^2^). Asthmatic and nonasthmatic subjects were studied based on BMI, (1) normal BMI (20–24.9 kg/m^2^) and (2) obese BMI (>30 kg/m^2^) to maximize potential differences on cys-LT synthesis. Stable asthmatics without exacerbations for 2 months were included. Diagnosis of asthma was based on history, physical exam, and pulmonary function testing including peak expiratory flow rate (PEFR) (L/min) (AsthmaCheck, Respironics, Inc., Youngwood, PA) performed within 3 months of starting the study. Approximately 50% of the asthmatics had spirometry, with forced expiratory volume in one-second (FEV_1_) measurements. Patients with mild to moderate persistent asthma (Steps 1–3) were studied. Subjects on inhaled corticosteroids (ICS) were included [26]. Peak flow meter and BMI values were determined in all subjects on the day of the study. An asthma questionnaire was administered which includes frequency of day and night time asthma symptoms [27].

Exclusion criteria included current smokers within the past 6 months and previous smokers > 20 pack years, intercurrent infection, and treatment with LT-modifier drugs, aspirin, nonsteroidal anti-inflammatory agents, and oral steroids. Aspirin sensitive asthmatics were excluded because of their tendency to display excess cys-LT synthesis at baseline [28].

### 2.2. Exhaled Breath Condensate

Exhaled breath condensate (EBC) was collected with RTube to determine airway lining fluid levels of cys-LT levels [29, 30]. The subjects breathe at their normal rate and depth for 5–7 min. The exhaled air cools below the dew point by transfer of heat to a chilled condenser surface. Condensation of the aerosolized airway lining fluid occurs as EBC. Samples were frozen at −70°C to be studied in batches later. cys-LT were determined by enzyme immunoassay (EIA) (Cayman Chemicals, Ann Arbor, MI). Samples were run in duplicate, neat, and diluted, using a competitive assay based on a high-affinity monoclonal antibody measuring cys-LT. The lower range detection limit (80%  *B*/*B*_0_) was 34 pg/mL. Significant numbers of both male and female patients were studied because of possible greater levels of leptin [31] and LT in women than in men [32].

### 2.3. Blood

A volume of 5 mL of heparinized whole blood was layered over 3.5 mL of a mixture of sodium metrizoate and Ficoll (1-Step Polymorphs, Accurate Chemical & Scientific Corporation, Westbury, NY) in a 15 mL centrifuge tube. The sample was centrifuged at 450 ×g for 30 min in a swing-out rotor at 22°C. Adipokines, leptin, and adiponectin were measured in the plasma by enzyme immunoassay (EIA) according to the protocol described [33]. Plasma leptin levels were determined spectrophotometrically using commercially available colorimetric EIA kits (Millipore, MA USA) and run according to the manufacturer's instructions. The coefficient of variation for duplicate samples was 3.7% and the lower limit of detection was 0.5 ng/mL. Similarly, plasma high molecular weight adiponectin (Linco Research, a subsidiary of Millipore, St. Charles, Mo) was run in duplicate according to the manufacturer's directions (LLD: 8.1%, 0.5 ng/mL.). Blood samples were drawn from patients at the same time of the day, in the morning, and when patients were in the fasting state. Total IgE levels were determined in all subjects (Clinical Pathology Laboratory, University of Michigan).

*Peripheral Blood Monocytes (PBM)*. We measured LT levels in PBM from subjects obese and nonobese with and without asthma. PBM were isolated from whole blood by Ficoll (One Step, Accurate Chemical & Scientific Corp, Westbury, NY) centrifugation and adherence, as previously described [34]. Cells were >90% PBM by differential staining and viability >95% by trypan blue exclusion. Isolated PBM were resuspended in lipopolysaccharide-free Dulbecco's Modified Eagle Medium at 0.5 × 10^6^/mL and adhered for 1 h at 37°C in a humidified atmosphere of 5% CO_2_/95% O_2_ for EIA. Nonadherent cells were removed by washing twice with Dulbecco's Modified Eagle Medium.

The cells were stimulated* ex vivo* with 1 *μ*M calcium ionophore A23187 (Calbiochem-Behring Corp., La Jolla, CA) in dimethyl sulfoxide [35]. Final concentrations of dimethyl sulfoxide (0.05%) added to cultures had no effect on either cell viability or eicosanoid synthesis. This compound liberates intracellular calcium from intact cells and releases arachidonic acid from cell membranes. It also activates lipoxygenases and stimulates LTC_4_ synthase. The eicosanoid products were measured by EIA.

### 2.4. Statistics

Our study was designed to have 80% power with a type I error 5% to detect significant differences between asthmatics and normal subjects, as well as between obese and nonobese asthmatics. From the preliminary data, we estimate the median log LTB_4_ levels in exhaled breath condensate for nonobese asthmatics as 50 pg/mL, corresponding to a median log level of 3.90. The study is powered to detect a doubling in the median LT levels for the obese compared to the nonobese groups. Based on preliminary data and to achieve the above differences 20 subjects were needed in each group, that is, 80 total for the four groups: obese asthma, nonobese nonasthma, asthma nonobese, and obese nonasthma.

Mean and standard deviations for normally distributed data were calculated and differences between groups determined by Student's *t*-test. For skewed data Mann-Whitney tests were utilized. Correlations and partial correlations were performed using Pearson's coefficient. For the evaluation of possible associations between each study variable (dependent) and BMI (independent), linear regression was performed. We log-transformed the data since it was not normally distributed. This log transformation resulted in normalization of the data. Multivariable regression analysis was performed for leptin, adiponectin, and EBC cys-LT levels, adjusting for age, gender, and asthma status. We evaluated the association with BMI categories as well as BMI as a continuous variable.

Comparison of study variables among the four groups was performed with one-way analysis of variance (ANOVA). The data were log transformed prior to ANOVA. *p* values < 0.05 were considered statistically significant. All statistics were performed on IBM SPSS version 21 statistical software.

## 3. Results

We set out to study four groups of subjects: obese asthmatics, nonobese asthmatics, obese nonasthmatics, and nonobese nonasthmatics. The total number of subjects studied was 82. By design the BMI of the control and obese subjects were recruited to obtain optimal separation of the groups (Table 1). Mean peak flow meter (PFM) readings of the asthmatics and nonasthmatics were also significantly different (Table 1). There was no difference in PFM between obese and nonobese (82.5 ± 21 versus 80.3 ± 12.2 L/min, *p* = ns) asthma subjects. There was also no significant difference in FEV_1_ between obese and nonobese asthmatics (74.6 ± 19.4 versus 91.5 ± 12.5% predicted, *p* = 0.052). The asthma control test (ACT) questionnaire was lower in obese asthma (19.8 ± 11.9) compared to nonobese asthma (22.1 ± 1.6) patients, *p* = 0.01. Total IgE levels were different but quite variable in both asthmatics and nonasthmatics (326 ± 519 versus 73 ± 124 kU/L, *p* = 0.004). There was no difference in IgE levels between obese and nonobese asthmatics (315 ± 448 versus 336 ± 586 kU/L, *p* = 0.13).

The gender breakdown was 64% female (36% male), which was as close as we could get to equality in recruitment. Males (35.2 ± 12.2 years) and females (35.1 ± 12.7 years) displayed no difference in age. No significant difference occurred in BMI between the genders, obese (36.5 ± 5.7 versus 38.4 ± 4.6 years male : female) or nonobese (23.6 ± 1.3 versus 22.5 ± 1.7).

The recruitment age in the subjects, asthma versus nonasthma subjects, was not significantly different (32.7 ± 12.3 versus 37 ± 12.1 years, *p* = 0.31). Obese subjects were generally older, displaying an age-related increase in fat mass (obese 41.8 ± 11.6 versus nonobese 27.7 ± 8.4,  *p* < 0.001). Obese asthmatics were on average older than nonobese asthmatics (40.6 ± 12.8 versus 25.5 ± 5.9 years, *p* < 0.001).

*Comorbidities*. There are a number of comorbidities that contribute to exacerbations of asthma. Twelve of the obese subjects had a diagnosis of obstructive sleep apnea (OSA). These were evenly divided among asthmatics and nonasthmatics (7/42 versus 5/40 subjects). Gastroesophageal reflux disease (GERD) was more common in obese than nonobese (8/42 versus 3/40 subjects) subjects. GERD was equally distributed between asthmatics and nonasthmatics (6/42 versus 5/40 subjects). 

*Medications*. The asthmatics studied had mild to moderate persistent disease with all of them on ICS (42 subjects). None of the asthmatics were on leukotriene modifier therapy, since that was an exclusionary criterion for subject recruitment.

### 3.1. Leptin

As expected, fasting leptin levels were higher in obese subjects than nonobese subjects (32.7 ± 20.2 versus 8.5 ± 6.3 ng/mL, *p* = 0.0001). There was no significant difference in leptin levels between asthmatics and nonasthmatics (Table 2). Mean leptin levels were higher in females than in males (26.4 ± 21 versus 12.4 ± 12.3 ng/mL, *p* < 0.05). Female asthmatics had higher leptin levels than males with asthma (31.6 ± 24.1 compared to 13.1 ± 11.9 ng/mL, *p* = 0.01). Leptin levels were higher in obese female asthmatics compared to obese male asthmatics (52.1 ± 18.6 versus 20.8 ± 11.6 ng/mL, *p* < 0.001). Leptin levels in female asthmatics were 31.6 ± 24.1 compared to females without asthma 20.9 ± 3.7 ng/mL, *p* = 0.03. Leptin levels in men with and without asthma were 13.1 ± 11.9 and 11.7 ± 8.5 ng/mL, respectively (*p* = 0.29).

Leptin's correlation with weight is 0.77 which was significant (*p* < 0.0001). When it was adjusted for asthma, it is 0.78 (*p* < 0.0001). The correlation of leptin with asthma is 0.17 (*p* = 0.119). When adjusted for BMI, the correlation was 0.27 (*p* = 0.14).

Linear regression demonstrated a positive correlation between leptin and obesity. There was also a positive correlation between leptin and the female gender (*p* = 0.001). Using multiple linear stepwise regression only weight and gender (female) had a positive correlation, when the variables age, gender, weight, and asthma were considered. There was no correlation between leptin and ICS or OSA.

**Figure 1:**
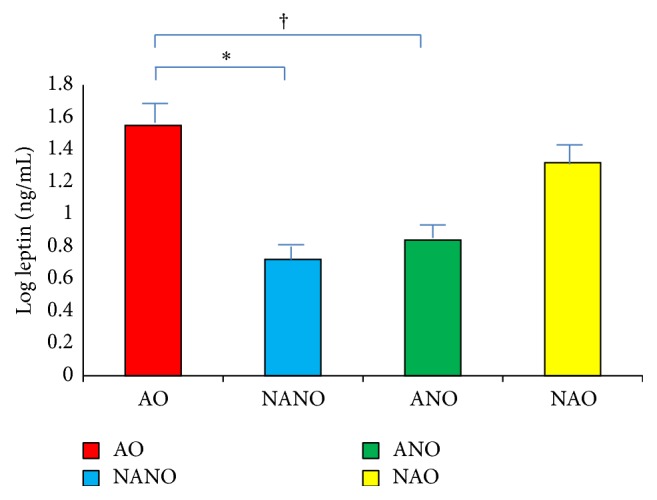
Increase in leptin levels in obese asthmatics. Serum leptin levels log mean transformed (ng/mL) in subjects with asthma obesity (AO), nonasthma nonobesity (NANO), asthma nonobesity (ANO), and nonasthma obesity (NAO). AO had significantly higher leptin levels (ANOVA *p* < 0.05), compared with NANO^*∗*^ and ANO^†^.

Using ANOVA, leptin was significantly different between the four groups: obese asthmatics, nonasthma nonobese, asthma nonobese, and nonasthma obese. Obese asthmatics had significantly higher levels than both nonobese asthmatics and nonobese nonasthmatics (*p* < 0.0001) (Figure 1). The only groups that were not statistically different were the obese asthmatics and obese nonasthmatics (*p* = 0.27).

### 3.2. Adiponectin

Adiponectin was decreased overall in the obese versus nonobese subjects (mean 9.3 ± 4.7 versus 14.1 ± 20 *μ*g/mL, *p* < 0.05), as expected. There was no difference in adiponectin levels between asthmatics compared to nonasthmatics (Table 2). As noted for leptin, mean adiponectin levels were higher in females than in males (14.1 ± 5.5 versus 7.2 ± 3.1 *μ*g/mL, *p* < 0.05) especially in nonobese. This was also true for obese females who had higher mean levels compared to obese males (11.3 ± 4.8 versus 6.4 ± 2.7 *μ*g/mL, *p* < 0.05). Males with asthma display lower mean levels of adiponectin than those seen in females with asthma (6.9 ± 2.8 versus 13.3 ± 5.6 *μ*g/mL *p* < 0.05).

Adiponectin's correlation with weight is −0.44 which was significant (*p* < 0.0001). When adjusted for asthma it was −0.44 (*p* < 0.0001). The correlation of adiponectin with asthma was −0.10 but was not significant (*p* = 0.36). When adjusted for BMI the correlation was −0.134 (*p* = 0.22). There was a positive correlation between adiponectin and ACT (0.35) which did reach significance (*p* = 0.002). When adjusted for asthma it was a positive correlation (0.36) and was significant (*p* = 0.001).

Linear regression demonstrated a negative correlation between adiponectin and obesity. Using multiple linear stepwise regression, there was a negative correlation with weight and a positive correlation with gender when the variables age, gender, weight, and asthma were considered. There was no correlation between adiponectin and ICS or OSA.

**Figure 2:**
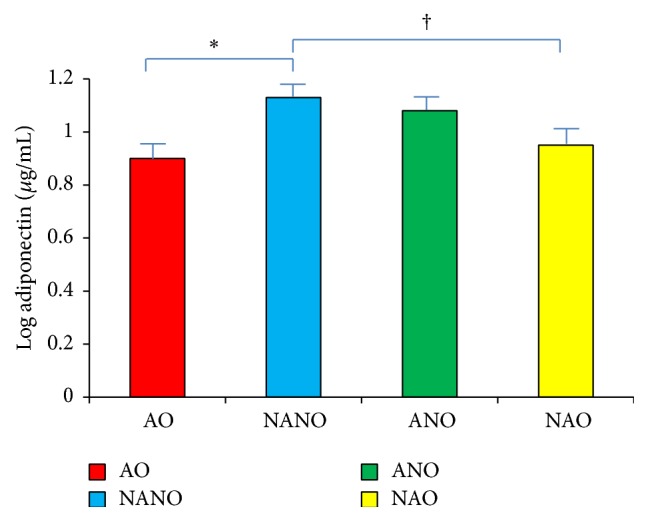
Lower adiponectin levels in obese asthmatics. Serum adiponectin levels log mean transformed (*μ*g/mL) in subjects with asthma obesity (AO), nonasthma nonobesity (NANO), asthma nonobesity (ANO), and nonasthma obesity (NAO). AO had significantly lower adiponectin levels (ANOVA *p* < 0.05) compared with NANO^*∗*^. NAO also had significantly lower adiponectin than NANO^†^.

ANOVA demonstrated that adiponectin was significantly different between the four groups: obese asthmatics, nonasthma nonobese, asthma nonobese, and nonasthma obese. Obese asthmatics had lower adiponectin levels than nonobese nonasthmatics (*p* < 0.008) (Figure 2). Obese nonasthmatics also had lower adiponectin than nonobese nonasthmatics (*p* = 0.017).

### 3.3. Leptin/Adiponectin Ratio

Since both leptin and adiponectin are increased in women compared to men, we next examined the leptin/adiponectin ratio as a method to better compare genders with regard to the role of adipokines in the pathogenesis of asthma in obesity. The mean leptin/adiponectin ratio was 2.8 ± 3.6 in women compared to 2.1 ± 1.9 in men (*p* = 0.15). Obese subjects had a significantly higher mean ratio than nonobese subjects (4.3 ± 3.5 versus 0.78 ± 0.8, *p* < 0.01). Asthmatics did not have a significantly higher mean ratio compared to nonasthmatics (Table 2). Although the mean leptin/adiponectin ratio trended to be higher in patients with worse airway obstruction (PFR < 80% predicted), it did not reach statistical significance (*p* = 0.26).

Linear regression demonstrated a positive correlation between leptin/adiponectin ratio and weight (*p* = 0.0001), as well as age (*p* = 0.0001). Using multiple linear stepwise regression, there was a correlation with weight (*p* = 0.0001). There was no correlation with gender, asthma, ICS, or OSA.

**Figure 3:**
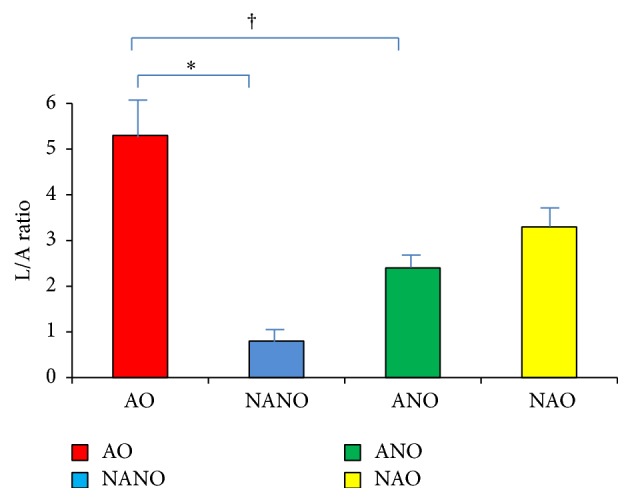
Elevated leptin/adiponectin ratio in obese asthmatics. Serum leptin/adiponectin (L/A) ratio in subjects with asthma obesity (AO), nonasthma nonobesity (NANO), asthma nonobesity (ANO), and nonasthma obesity (NAO). AO had significantly higher L/A ratio (ANOVA *p* < 0.05) compared with NANO^*∗*^ and ANO^†^.

ANOVA demonstrated that leptin/adiponectin ratio was significantly different between the four groups: obese asthmatics, nonasthma nonobese, asthma nonobese, and nonasthma obese. Obese asthmatics had higher leptin/adiponectin ratios than nonobese nonasthmatics (5.3 ± 4.3 versus 0.75 ± 0.9, *p* < 0.0001) and nonobese asthmatics (*p* < 0.0001) (Figure 3), but not obese nonasthmatics (*p* = 0.16). This was mainly accounted for by female obese asthmatics (6.3 ± 4.9 versus 3.3 ± 1.7 for males, *p* = 0.03).

### 3.4. EBC cys-LT Levels

Exhaled breath concentrate (EBC) cys-LT levels were higher in all asthmatics compared to nonasthmatics (Table 2). There was no difference in EBC cys-LT levels between obese and nonobese subjects (66.1 ± 29.00 versus 62.8 ± 29.1 *μ*g/mL, *p* = 0.3). Obese asthmatics had higher EBC cys-LT levels than nonobese nonasthmatic subjects (73 ± 29.7 versus 57 ± 28.1 *μ*g/mL, *p* = 0.05).

The correlation of EBC with obesity was 0.11 (*p* = 0.34). When it was adjusted for asthma it was 0.13 (*p* = 0.26). EBC correlation with asthma was 0.23 which is significant (*p* = 0.04). When this is adjusted for BMI it was 0.24 (*p* = 0.03).

Multiple regression analysis revealed a significant correlation between EBC cys-LT and asthma (*p* = 0.04). There was no correlation with weight, age, gender, ICS, or OSA.

**Figure 4:**
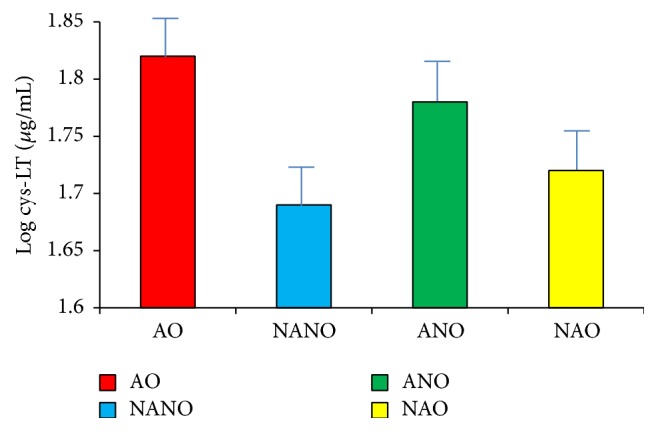
Exhaled breath condensate cysteinyl leukotriene levels in obesity and asthma. Exhaled breath condensate (EBC) cys-LT levels log mean transformed (*μ*g/mL) in subjects with asthma obesity (AO), nonasthma nonobesity (NANO), asthma nonobesity (ANO), and nonasthma obesity (NAO). There was no significant difference between the groups with ANOVA testing.

ANOVA demonstrated no difference between four groups, likely due to the variability of the samples. The greatest difference was between obese asthmatics and nonobese nonasthmatics but only reached a *p* value of 0.3 (Figure 4).

### 3.5. Peripheral Blood cys-LT

PBM cys-LT production was higher in females than males (222 ± 132 versus 142 ± 116 *μ*g/mL, *p* < 0.05). PBM cys-LT levels were comparable in the asthmatic versus nonasthma subjects (197 ± 148 versus 190 ± 115 *μ*g/mL, *p* = 0.4). However, in view of the increased variability of the PBM cys-LT levels, there were no significant increased values in obese asthmatics compared to nonobese nonasthmatics.

## 4. Discussion

The main findings of this study are the following: (1) plasma leptin levels are increased in obese asthmatics compared to obese nonasthmatics. This was mainly accounted for by higher leptin levels in females, and especially obese female asthmatics. (2) Adiponectin levels were lower in obese asthmatics compared to nonobese nonasthmatics. (3) The leptin/adiponectin ratio was significantly higher in obese asthmatics compared to nonobese nonasthmatics. (4) EBC cys-LT levels were higher in asthmatics than in nonasthmatics. (5) PBM cys-LT levels were higher in women than in men, irrespective of asthma or obesity.

An important finding in our study was the association between leptin and asthma, and specifically in obese asthmatics. This was especially true in obese female asthmatics. Leptin levels are thought to be higher in females because of increased percentage body fat in women [31] as well as increased secretion of leptin from adipose tissue. The latter may be related to increased estradiol levels and its effect of increased leptin secretion from adipose tissue [36]. Leptin has been noted to be a proinflammatory adipokine driving the immune system towards a predominant Th1 phenotype [37]. Leptin may also have an impact on the airway milieu in asthmatics with obesity. Leptin may increase proinflammatory phenotype in macrophages [38], augmenting the response to LPS. Leptin receptors have been described in the airway and investigators have shown a correlation between plasma and BAL levels [38]. Leptin deficient mice display reduced LT synthesis, and exogenous leptin augments LT production in macrophages from these animals [19]. Obesity has also been associated with an alteration in adipokines that predispose to neutrophilic inflammation [39]. However, other investigators have demonstrated decreased inflammation in the airways of obese asthmatics [40], which is reversed by weight loss. They propose that adipokines may augment airway bronchial reactivity without increasing airway inflammation. Recently, it has also been shown that leptin may also affect the airway diameter through noninflammatory pathways by inhibiting the cholinergic pathway [22]. Although we have shown an association between leptin and asthma, population studies by Sutherland et al. have shown no such association [41]. Sampling of nonfasting subjects, some of whom smoked, can affect leptin levels and may explain the differences in conclusions of these studies.

Adiponectin is another adipokine formed in adipose tissue. In contrast to leptin, adiponectin levels are decreased in obesity and tend to increase in starvation. It has a number of metabolic actions including increasing insulin sensitivity, diminishing atherosclerosis, and being generally anti-inflammatory [24]. Although adiponectin receptors have been detected in the lungs, serum levels do not consistently correlate with BAL fluid levels. This finding may be explained by the fact that active transport of adiponectin, which is a large molecule (full length form), may be necessary for penetration into the airways [42]. Higher adiponectin levels have been associated with lower asthma prevalence rates in women [43]. In our population, obese asthmatics had lower adiponectin levels than nonobese nonasthmatics. We also demonstrated a positive correlation between adiponectin and asthma symptoms (ACT). However, the higher levels of adiponectin in women compared to men, as was noted for leptin, may be the confounding variable, which detracts from the association of lower adiponectin levels in obese asthmatics compared to other groups. Other investigators have questioned the association between adiponectin and asthma. Cross-sectional analysis of an asthmatic population, who were nonfasting and smokers, did not demonstrate an association between adiponectin and asthma [41].

An increased leptin/adiponectin ratio is associated with asthma compared to control subjects without asthma. The increased leptin/adiponectin ratio is also associated with severe asthma compared to mild to moderate asthma [44]. In our study, leptin/adiponectin ratios were significantly elevated in obese asthmatics compared to nonobese nonasthmatics. Obese asthmatic women had the highest leptin/adiponectin ratio. However, in our study the leptin/adiponectin ratio was not associated with asthma or increased airway obstruction.

The reduced responsiveness to inhaled corticosteroids with increasing BMI, with a relatively stable response to montelukast, has been previously described [15]. Sutherland et al. also examined the comparative effects of BMI in response to asthma controller medication [45]. By contrast, they demonstrated that inhaled corticosteroids were superior to LT modifiers at all BMI levels, in patients who were underweight compared to those with morbid obesity. In our study we demonstrated that EBC cys-LT levels correlated with asthma, being higher in asthmatics than in nonasthmatics. The trend was greater in obese asthmatics compared to nonobese asthmatics. Other investigators have demonstrated an association between BMI and urine cys-LT in obese asthmatics [46]. Obese patients had significantly higher values of LTE_4_/creatinine in urine compared to subjects who were preobese and in the normal range. In a linear regression model, the only significant associations were those between BMI and LTE_4_/creatinine in urine. Using the same model, log leptin and log adiponectin presented positive and negative associations, respectively, with LTE_4_/creatinine in urine. Notably, there was no association between BMI and EBC LTE_4_ levels [46]. This was because of the increased variability noted with EBC samples, which may also explain the marginal increase in EBC cys-LT levels in obese asthmatics in our study. The other problem is the inability to adequately standardize dilution factors for EBC across subjects. An alternative explanation is that alveolar cys-LT synthesis may not be as important as airway levels. A recent study demonstrated elevated submucosal but not sputum eosinophil levels in obese subjects with severe asthma [47, 48]. This may explain the elevated urine cys-LT levels in obese subjects compared to EBC measurements [46].

In our cohort subjects with the older obese women phenotype trended to have increased leptin and EBC cys-LT levels with worse pulmonary function. Obese female asthmatics had higher leptin levels than obese male asthmatics. Recently, investigators have demonstrated that BMI was associated with the incidence of asthma in women but not in men [49]. Furthermore, adult onset nonatopic asthma has become the most common type of asthma in women [50]. Investigators have demonstrated that both testosterone (negative) and estrogen (positive) were associated with alterations in adipokines, specifically leptin levels [51]. We detected no significant difference between premenopausal obese women with asthma compared to nonobese patients. There was no decline in leptin levels in asthma with age, in postmenopausal compared to premenopausal subjects matched for BMI. Similarly, there was no change in adiponectin levels. The increase in BMI with age may have offset the effect of any change in estrogen/testosterone levels on adipokine levels as subjects increased in years. We did document that PBM from female subjects had higher cys-LT levels than that of male subjects. This has been noted by other investigators and may in part be due to suppression of 5-LO enzymatic activity by testosterone [52]. Although PBM cys-LT levels were trending higher in asthmatics than nonasthmatics, this did not reach significance. Increased plasma leptin levels in women may be associated with the elevated PBM cys-LT production noted in females [20].

We documented comorbidities of OSA and GERD in asthmatics and nonasthmatics. Poorly controlled OSA has been shown to worsen asthma control, in part through increased release of proinflammatory mediators during episodes of hypoxia and disrupted sleep [53]. Nocturnal GERD may exacerbate asthma with aspiration of acid, during inhalation against a closed airway, following episodes of upper airway obstruction. Like OSA, the incidence of GERD is also increased in obese subjects and may exacerbate airway disease after eating. GERD can result in reflex bronchospasm from irritation of the esophagus or directly spilling into the airway with gross reflux. Although both OSA and GERD were more common in obese than nonobese subjects in our study, the incidence was equally distributed in the asthmatic and nonasthmatic groups.

There are a number of potential limitations with this study. It is a relatively small study number-wise, examining the role of obesity in the pathogenesis of asthma. Furthermore, it is an observational study, with no interventions. Although the diagnosis of asthma was made by a physician with history, physical examination, and pulmonary function testing, spirometry or methacholine challenge testing was not performed. Spirometry with FEV_1_ measurements was only obtained in ~50% of the asthma subjects. Instead PFM readings were obtained during the study visit. In addition, a number of variables to assess the role of EBC cys-LT readings limited the interpretation of results. Inhaled corticosteroids can result in ~18% reduction in EBC cys-LT levels, but we wanted to include asthmatics that were with mild to moderate severity [54]. Although adipokines can be affected by the gender and the menstrual cycle, we did not determine the reproductive hormone concentrations in our premenstrual patients. Despite these issues, the study has a number of strengths. In recruiting subjects, there was good characterization and separation of subjects between the control group and obese subject group. In addition, the asthmatics were well characterized compared to the nonasthmatics with peak flow rates and asthma control test questionnaires. Furthermore, the significance of the study was improved since we studied predominantly moderate persistent asthmatics all on inhaled corticosteroid therapy. None of the asthmatics were on LT modifier medications.

In summary, we have demonstrated that leptin levels and the leptin/adiponectin ratio are increased in obese asthmatics compared to obese nonasthmatics. Proinflammatory mediators, cys-LT, were increased in obese asthmatics compared to nonobese nonasthmatics. Adipokines, especially in obese female asthmatics, may promote an asthma phenotype that has become more prevalent and results in difficult to control airway disease.

## Figures and Tables

**Table 1:** Demographics of the study population of obese, nonobese, asthmatic, and nonasthmatic subjects.

	Asthma	Nonasthma	*p*

*n*	42	40	
BMI	30.3 ± 8.6	30.4 ± 7.9	= 0.5
PFR L/min	82 ± 17.6	107 ± 16.3	= 0.01
M/F	15/27	15/25	

Subgroups			
	Obese/nonobese	Obese/nonobese	

*n*	21/21	21/19	
BMI	38.7 ± 5.4/23.0 ± 1.8	37.1 ± 3.6/23.1 ± 1.8	
PFR L/min	82.5 ± 21/80.3 ± 12.2	91.5 ± 14.2/89 ± 18.9	
M/F	8 : 13/7 : 14	10 : 11/5 : 14	

Note: M/F: male/female; PFR: peak flow rate % normal predicted; BMI: body mass index.

**Table 2:** Adipokine levels in obese, nonobese, asthmatic, and nonasthmatic subjects.

	Asthma	Nonasthma	*p*
Leptin ng/mL	24.9 ± 22.3	17.4 ± 15.3	= 0.125
Adiponectin *μ*g/mL	10.9 ± 5.7	12.3 ± 5.9	= 0.13
L/A ratio	3.0 ± 3.8	2.1 ± 2.2	= 0.09
EBC *μ*g/mL	70.1 ± 29.4	59.1 ± 27.8	= 0.04

Subgroups			
	Obese/nonobese	Obese/nonobese	

Leptin ng/mL	40.2 ± 21.9/9.7 ± 7.2	26.8 ± 15.8/7.1 ± 4.5	
Adiponectin *μ*g/mL	9.0 ± 4.7/12.6 ± 6.3	9.5 ± 4.9/14.7 ± 5.6	
L/A ratio	5.5 ± 4.3/2.5 ± 0.76	2.3 ± 2.1/3.1 ± 0.3	
EBC *μ*g/mL	72.9 ± 29.7/67.8 ± 29.7	60.8 ± 28.1/57.1 ± 28.2	

Note: L/A: leptin/adiponectin ratio; EBC: exhaled breath condensate.
